# Hydrogen Sulfide: Potent Regulator of Vascular Tone and Stimulator of Angiogenesis

**Published:** 2012-06

**Authors:** Carolin Köhn, Galyna Dubrovska, Yu Huang, Maik Gollasch

**Affiliations:** 1 *Medical Clinic for Nephrology and Internal Intensive Care, Charité Campus Virchow Klinikum and Experimental and Clinical Research Center (ECRC), Max-Delbrück Center for Molecular Medicine, Berlin, Germany;*; 2 *Institute of Vascular Medicine, Li Ka Shing Institute of Health Sciences, and School of Biomedical Sciences, Chinese University of Hong Kong, Shatin, N.T., Hong Kong, China*

**Keywords:** adipocyte-derived relaxing factor, ADRF, KCNQ channels, K_v_7 channels, periadventitial vasoregulation, H_2_S

## Abstract

Hydrogen sulfide is the “third” gasotransmitter on the rise in cardiovascular research. Recent studies show that hydrogen sulfide has a great potential in the regulation of vascular tone of systemic arteries and many molecular targets are discussed. However, the complex mechanism of vascular tone regulation by hydrogen sulfide is only incompletely understood. It seems that a potent interaction of hydrogen sulfide with vascular endothelial growth factor (VEGF) becomes important in angiogenesis, in the process of wound healing, but also in tumor angiogenesis. Hydrogen sulfide exerts anti-inflammatory effects and it could be a pharmacological target in vascular dysfunction in association with obesity-related hypertension as well as in tumor development and progression. However, the underlying molecular pathways still need to be revealed. This review primarily focuses on the regulatory role of hydrogen sulfide in controlling vascular tone. We attempt to provide recent insights into mechanisms by which CSE-dependent hydrogen sulfide plays a role in the regulation of vascular tone by perivascular adipose tissue. The role of KCNQ channels and other ionic permeation pathways as key targets will be discussed. Recent findings which are summarized in this paper provide new insights into molecular mechanisms of hydrogen sulfide that are crucial for understanding vascular dysfunction in cardiovascular disease and possibly angiogenesis. Future research will be extended to investigate the therapeutic potential of hydrogen sulfide and their targets such as KCNQ channels in cardiovascular diseases, angiogenesis and tumor genesis.

## INTRODUCTION

### Hydrogen sulfide the “third” gasotransmitter

Hydrogen sulfide (H_2_S) is the “third” gasotransmitter next to nitric oxide (NO) and carbon monoxide (CO). A wide range of biological effects may be associated with H_2_S, including improved reperfusion in trauma and circulatory shock (1), retardation of cardiac hypertrophy through its anti-fibrotic property (2), anti-hypertensive effects through vasorelaxation (1) and suppression of vascular inflammation (3). Furthermore, H_2_S regulates tumor angiogenesis via modulation of VEGF and activation of phosphatidylinositol-3-kinase (PI3K) and mitogen activated protein kinases (MAPK) (4).

**Figure 1:**
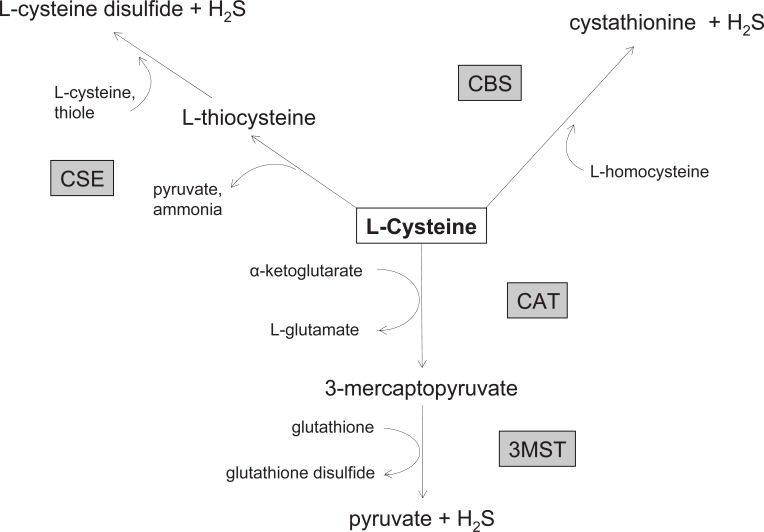
Biochemical and metabolic pathways leading to the production of hydrogen sulfide. CSE, cystathionine-γ-lyase; CBS, cystathionine-ß-synthase; CAT, cysteine-(aspartate)-aminotransferase; 3MST 3-mercaptopyruvate-sulfurtransferase. Figure modified from (8).

H_2_S is endogenously produced by cystathionine-γ-lyase (CSE) and cystathionine-ß-synthase (CBS) (1, 5). Other sources, particularly in red blood cells, have been identified (6), which suggest another H_2_S-generating enzyme, the 3-mercaptopyruvate-sulfurtransferase (3MST) (7, 8). H_2_S derived from CSE and CBS seems dependent upon pyridoxalphosphate (5) and the calcium calmodulin complex (9). Hypoxia may affect H_2_S production (10). In addition, tissue oxygen levels also regulate H_2_S-mediated actions (11). The different sources of H_2_S generation and biochemical pathways are presented in Figure 1 (modified from (7)).

Recent studies suggest a role for H_2_S in wound healing and angiogenesis. H_2_S promotes vessel formation via modulation of hypoxia inducible factor 1α (HIF-1α) and up-regulation of VEGF expression (12, 13). In addition, opening of K_ATP_ channels (14) and activation of phosphorylation cascades might be also involved. H_2_S activates phosphatidylinositol-3-kinase (PI3K) and mitogen activated protein kinases (MAPK) in endothelial cells (4, 14). CSE inhibition reduces the pro-angiogenic effect of H_2_S (4). Some studies show that H_2_S exerts its proangiogenic effect in tumor cells. Exogenous H_2_S increases cytosolic calcium levels and activates potassium currents to stimulate migration (15). CSE inhibition can reduce tumor pro-angiogenic signaling via decreasing VEGF (15).

## DISCUSSION

### Cystathionine-γ-lyase (CSE), H_2_S and ADRF

Soltis and Cassis showed that perivascular adipose tissue attenuated the contractile response of rat aorta to norepinephrine (16). Perivascular fat attenuated the contractile response of systemic arteries to serotonin, phenylephrine, angiotensin II, and other physiological relevant molecules (17, 18). Löhn *et al*. suggested that this anti-contractile effect is caused by an “adipocyte derived relaxing factor” (ADRF) which is yet to be identified (17). ADRF is a transferable factor that induces opening of voltage-dependent potassium (K_v_) channels in vascular smooth muscle cells (VSMCs) and thus leads to cell membrane hyperpolarisation (17, 18). Glibenclamide partially inhibits ADRF effects in rat aortas by non-specific effects, which are distinct from inhibiting K_ATP_ channels (19). ADRF is independent of the presence of endothelium and NO-mediated mechanisms (17). Leptin- and adenosine receptors do not participate in the anticontractile effect of perivascular fat (17). Inhibition of cyclooxygenase (COX) or cytochrome-P450-enzymes has no influence either (17). However, tyrosine kinases and PKA seem to be involved in the ADRF release (17, 20). ADRF secretion is calcium-dependent. Sodium channels and vanilloid/canniboid receptors have no role in the ADRF effect, thus minimizing the possible involvement of perivascular nerve activity (20).

**Figure 2:**
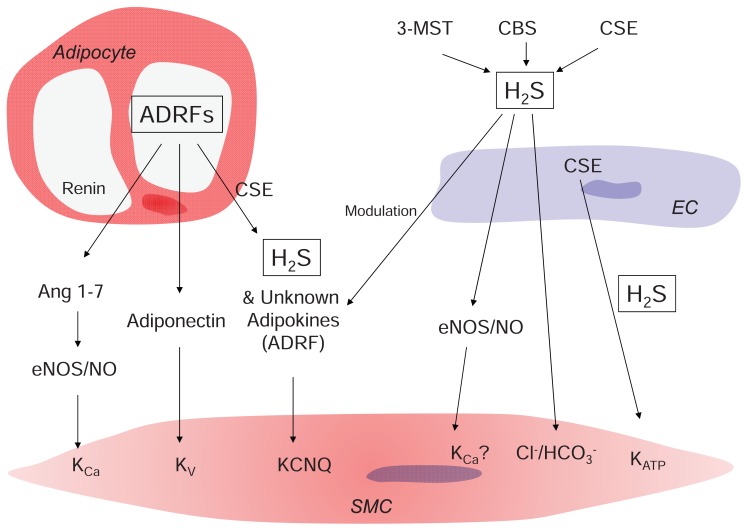
Putative mechanisms involved in the regulation of arterial vascular tone by ADRF, H_2_S, and other adipokines. NO, nitric oxide. NOS, NO synthase. K_Ca_, calcium-activated potassium channels. K_v_, voltage-dependent potassium channels. KCNQ, KCNQ channels. Cl^-^/HCO_3_^-^, Cl^-^/HCO_3_^-^exchanger. CSE, cystathionine-γ-lyase; CBS, cystathionine-ß-synthase; 3MST, 3-mercaptopyruvate-sulfurtransferase. SMC, vascular smooth muscle cell; EC, endothelial cell; Ang 1-7, angiotensin 1-7.

Several factors have been discussed as putative ADRFs. Renin-angiotensin system components such as angiotensin 1-7 (21) and CSE-derived H_2_S are promising ADRF candidates (Figure 2). However, the nature of ADRF remains elusive. Perivascular fat exerts a vaso-protective effect in mouse and rat systemic peripheral arteries, while such protection appears to be lacking in hypertension and obesity. New Zealand Obese (NZO) mice are commonly used in obesity research and their blood vessels are surrounded by increased amounts of perivascular fat. Interestingly, the vascular smooth muscle cells of very obese and diabetic NZO mice do not respond to paracrine modulation of perivascular fat via ADRF (22). Spontaneous hypertensive rats (SHR) show also diminished anticontractile effects of perivascular adipose tissue (23).

Recent data suggest that H_2_S plays an important role in tumor genesis and progression (24), which may in part be explained by inhibition of NF-kappaB and superoxide formation (3, 24-26). However, the complex molecular pathways and interactions between obesity, hydrogen sulfide, and perivascular fat are still poorly understood.

Our group has recently investigated the interaction and potential target molecules of ADRF and H_2_S by focusing on 1) CSE-dependent H_2_S regulation of vascular tone and 2) the possible role of KCNQ channels. Major results, together with recent studies on this topic, are presented in this paper.

### Role of CSE and KCNQ channels in periadventitial vasorelaxation

**Figure 3:**
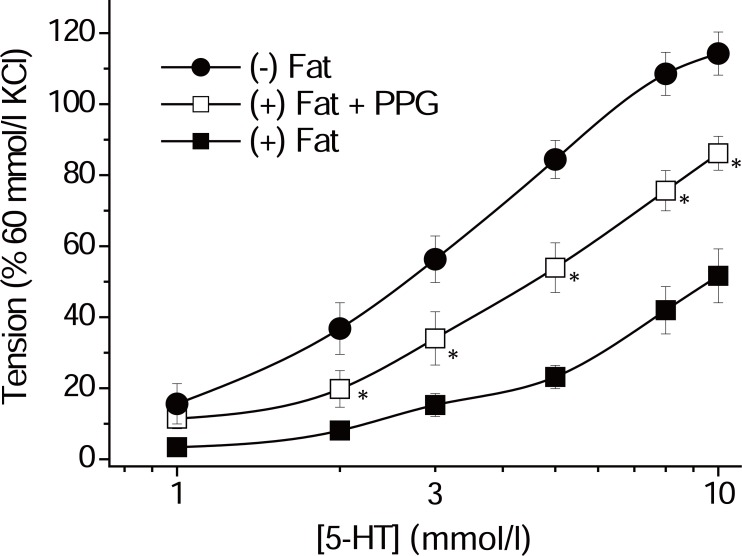
Serotonin (5-HT) dose response curve of rat aortic rings with and without perivascular fat with and without preincubation with PPG (30 min, 10 mmol/l). *p<0.05, n=9 in groups without preincubation. n=8 for groups with PPG. Isometric contractions of isolated male adult rat aortic vessels. Perivascular fat and connective tissue were either removed ((-) fat) or left intact ((+) fat) as previously described by Löhn *et al*. (17, 18, 20, 38). PPG had no effects on (-) fat rings (not shown). Tension is expressed as a percentage of the steady state tension (100%) obtained with isotonic external 60 mmol/l KCl (28). A value of p less than 0.05 was considered statistically significant; n represents the number of arteries tested.

Fang *et al*. and our group investigated the role of CSE-dependent H_2_S in the anticontractile effect of perivascular fat in rat aortas (27, 28). Rat aortic rings without perivascular fat exhibited a significantly stronger contraction in response to serotonin than rings with perivascular fat. CSE inhibition by PPG attenuated the anticontractile effect of perivascular fat (Figure 3) without affecting contractions in rings without perivascular fat (27, 28), suggesting that CSE-H_2_S may be involved in the anticontractile effects. Similar effects were seen with KCNQ channel inhibitor, XE991. XE991 attenuated the anticontractile effect of perivascular fat, suggesting that KCNQ channels mediate the ADRF effect, at least in part, in rat aortas (28).

### Relaxation of aortic rings by NaHS

H_2_S is a potent exogenous vasodilator. However, endothelial cells has been proposed to serve as endogenous source of H_2_S. A number of pathways have been suggested to play a role in vasorelaxation by H_2_S, including formation of cyclic adenosine-mono-phosphate (cAMP) and subsequent action of protein kinase A (PKA) (29), endogenous prostanoids (30), activation of ATP-dependent potassium channels (K_ATP_) and of the Cl^-^/HCO_3_^-^ transporter (11, 31, 32). Figure 2 illustrates these important pathways. However, at low concentrations H_2_S promotes vasoconstriction via inhibition of the cAMP pathway (29) and possibly by binding NO, forming an inactive nitrosothiol (33).

Our group tested the hypotheses that H_2_S can induce vasorelaxation by opening KCNQ channels in vascular smooth muscle cells (28). Serotonin precontracted rat aortic rings without perivascular fat were exposed to the H_2_S donor NaHS. One group of rings was preincubated with XE991 before precontraction with serotonin. The other groups of rings were preincubated with Kv inhibitor 4-AP or XE991 or K_ATP_ channel blocker glibenclamide, respectively. NaHS induced a larger vasorelaxation in precontracted aortic rings with fat. This relaxation was blocked by XE991 (28) and attenuated by glibenclamide and 4-AP (data not shown). These results indicate that exogenous H_2_S can induce stronger relaxation in rat aortas and this relaxation involves opening of KCNQ channels and/or cooperative opening of both KCNQ and K_ATP_ channels.

### Complex interaction between ADRF and H_2_S

The studies of Fang *et al*. and our studies provide novel insights into the complex interaction between ADRF and H_2_S (27, 28). First, H_2_S modulates vascular tone of rat aortas in a perivascular fat-dependent manner. CSE inhibition attenuates the anticontractile effect of perivascular fat in these arteries. Furthermore, exogenous H_2_S induces vasorelaxation which can be blocked by KCNQ channel inhibition. Glibenclamide is also effective, indicating involvement of K_ATP_ or non-specific effects as observed earlier by our group in this preparation (19). Thus, H_2_S might act as a putative ADRF targeting KCNQ channels, at least, in rat aortas.

Second, CSE-derived H_2_S is unlikely a direct ADRF candidate in mouse aortas as it is observed that CSE inhibition with PPG does not affect the anticontractile effects of perivascular fat (unpublished data). CBS is another enzyme catalyzing the formation of H_2_S. Many publications describe CSE and CBS as the main enzymes catalyzing the endogenous H_2_S production from l-cysteine (1). However, its effect in the regulation of vascular tone by perivascular fat is largely unclear. Future studies should determine whether inhibition of CBS is able to reduce the ADRF effect in mouse aortas. Further studies are also needed to investigate whether exogenous l-cysteine, the main precursor of endogenously produced H_2_S, is able to induce relaxation in mouse aortas and whether the effects can be blocked by CSE inhibition. Furthermore, a possible role of 3MST needs to be investigated to understand the complex interaction of endogenously produced H_2_S and perivascular fat (Figure 1).

### Activation of KCNQ channels by H_2_S (ADRF)

Based on pharmacological data with XE991, an inhibitor of KCNQ channels, our group was able to identify a specific role of KCNQ channels in inducing relaxation by perivascular adipose tissue and exogenous NaHS (H_2_S) in rat aortas. Thus, opening of KCNQ channels seems to be a key mechanism in vasorelaxation induced by both ADRF and H_2_S. The results suggest that KCNQ channel opening might also serve as a powerful mechanism to induce vasorelaxation in ADRF malfunctioning such as in obesity and hypertension. Future research needs to focus on this hypothesis.

CSE-derived H_2_S has been recently proposed as ADRF in rat aortas (27, 28). In line, Fang *et al*. showed CSE expression in vascular smooth muscle cells and periadventitial adipocytes but not in the endothelium of rat aortas (27). In contrast, Yang *et al*. detected CSE expression in endothelium but not in VSMCs in mouse aortas (9). In line with the latter results, we observed that CSE inhibition by PPG does not modify the relaxation by perivascular adipose tissue in mouse aortas. Taking together, CSE-derived H_2_S seems to be rather an ADRF modulator than a direct ADRF in mouse aortas (Figure 2). However, PPG had to be used at suspicious high concentrations in order to block CSE. More detailed studies using different CSE inhibitors, other donors than NaHS, and CSE deficient mice are essential to underpin the role of CSE in endothelial and periadventitial vasoregulation of arterial tone.

### Exogenous H_2_S donors and novel therapeutic strategies

As mentioned earlier, a number of studies reported strong vasorelaxation of systemic arteries by NaHS. However, the results with NaHS are difficult to interpret because of the unknown kinetics of H_2_S release by NaHS. ADTOH is a novel, promising H_2_S donor. ADTOH is a dithiole-3-thione moiety of H_2_S-releasing aspirin and therefore not only liberates H_2_S but also suppresses the thromboxane A_2_ activity (34). It will be important to test whether ADTOH mimics ADRF *in vitro* and *in vivo*. These studies will clarify the role of exogenous H_2_S in the ADRF effects and the complex interaction between inflammation, H_2_S and perivascular fat in vascular dysfunction of obesity-related hypertension.

Previous studies identified elevation of various pro-inflammatory cytokines secreted by perivascular adipose tissue in states of ischemia and hypoxia whereas protective effects of adiponectin and other adipokines were reduced. Involved molecules are components of the renin-angiotensin system, interleukin 1 (IL-1), IL-6, tumor necrosis factor α (TNF α), and C-reactive protein (35, 36). Additional production of reactive oxygen species increases oxidative stress which triggers vascular inflammation, associated with hypertension (37), and may promote cancer development (24). Kotsis *et al*. showed that malfunctioning adipose tissue in obesity not only stimulates the release of pro-inflammatory molecules but also that of thromboxane A_2_ (35). Therefore, studies using ADTOH might not only provide information about H_2_S as an ADRF modulator but also present a novel putative link between its vasodilatory and anti-inflammatory actions in states of ADRF malfunction. ADTOH and other H_2_S donors may provide novel therapeutic strategies to target ADRF malfunction. Therefore, this research is expected to identify novel, promising pharmaceutical strategies to treat vascular dysfunction in cardiovascular diseases.

## CONCLUSIONS AND PERSPECTIVES

Investigation of the paracrine role of adipose tissue in regulating vascular tone and function is an exciting and rapidly advancing area of medical research which provides many new and emerging pathophysiological links to cardiovascular diseases. Recent research is focused on the complex interaction between perivascular adipose tissue and H_2_S as novel regulators of vascular tone and modulators of vascular inflammation. However, future studies and genetic approaches are required to clarify the role of CSE in the vasculature. Recent research has identified KCNQ channel opening as a powerful mechanism to induce vasorelaxation by the mode of H_2_S-mediated regulation. However, this conclusion is mainly based on the use of the pharmacological blocker XE991 in rat and mouse aortas. Therefore, additional studies are required to determine the role of these channels in other vascular beds. Furthermore, we tend to believe that ADTOH could be a promising novel molecule to attenuate the progression of vascular dysfunction.

This manuscript describes vascular effects of H_2_S and perivascular adipose tissue and presents some new insights into putative pharmaceutical targets for the treatment of obesity-related hypertension. However, similar mechanisms of local vascular inflammation and hypoxia also play a crucial role in vessel formation, tumor development and growth so that these studies shall also provide a basis for further research in angiogenesis and cancer with a hope of finding new therapeutic strategies.
